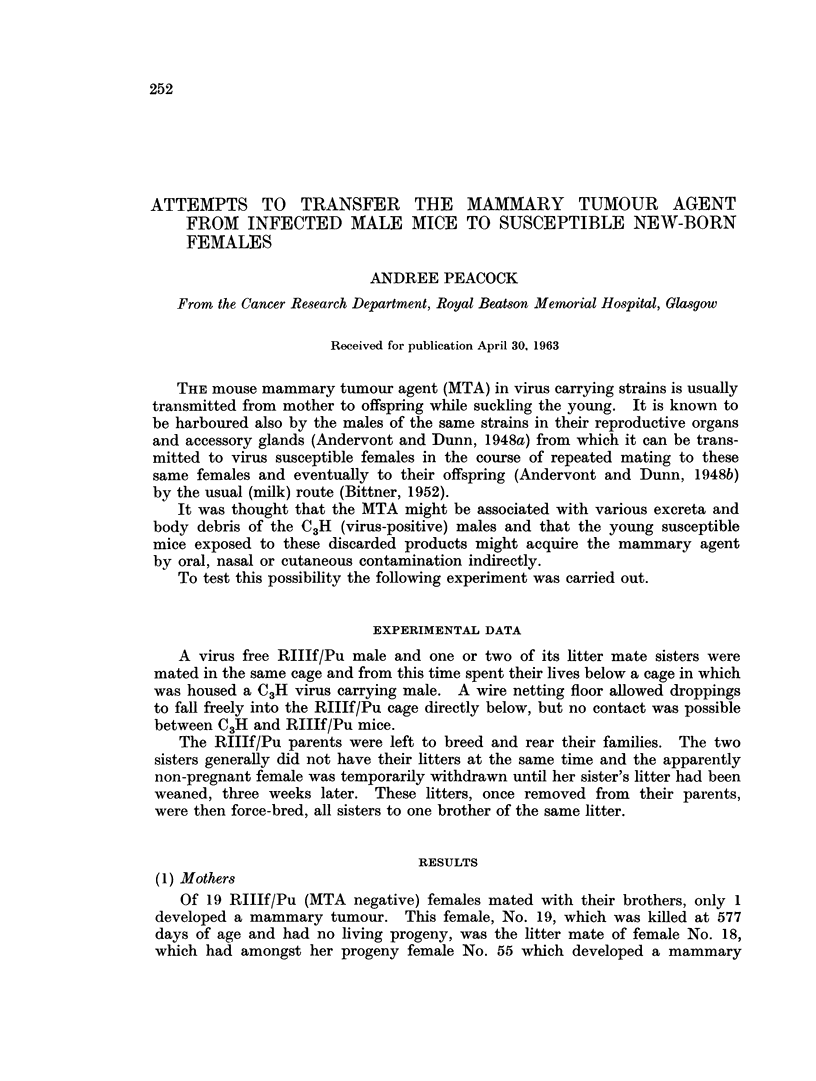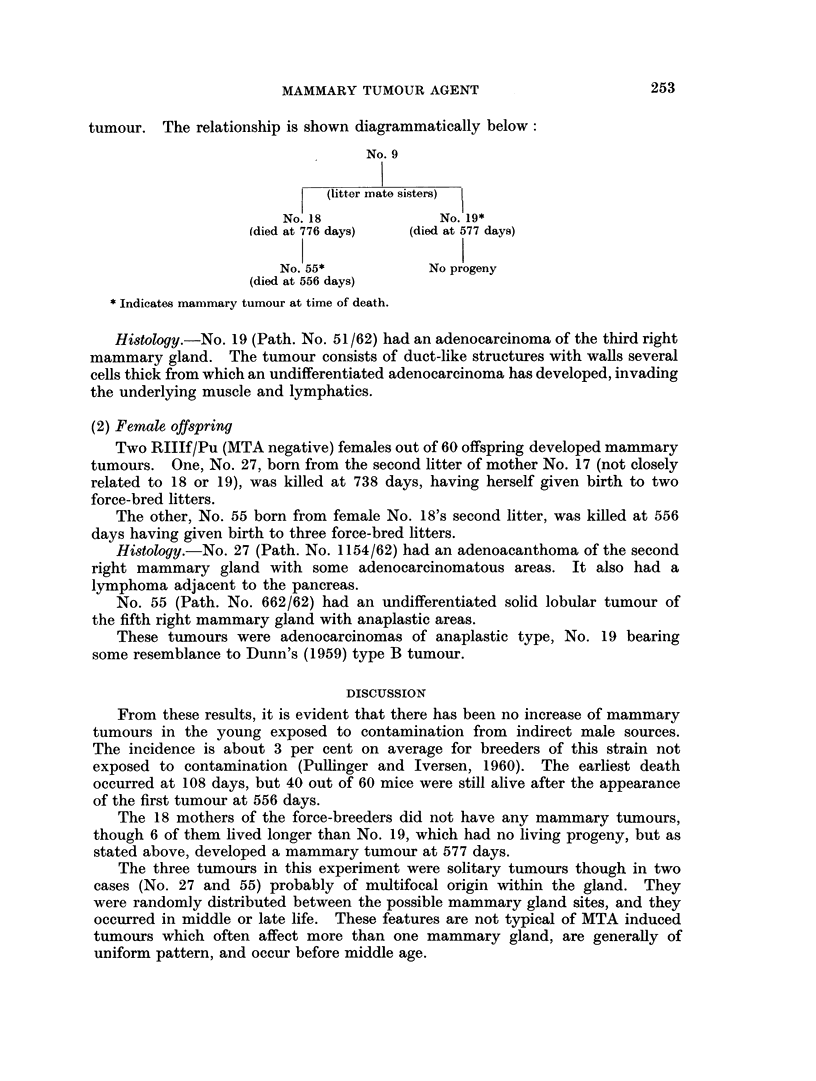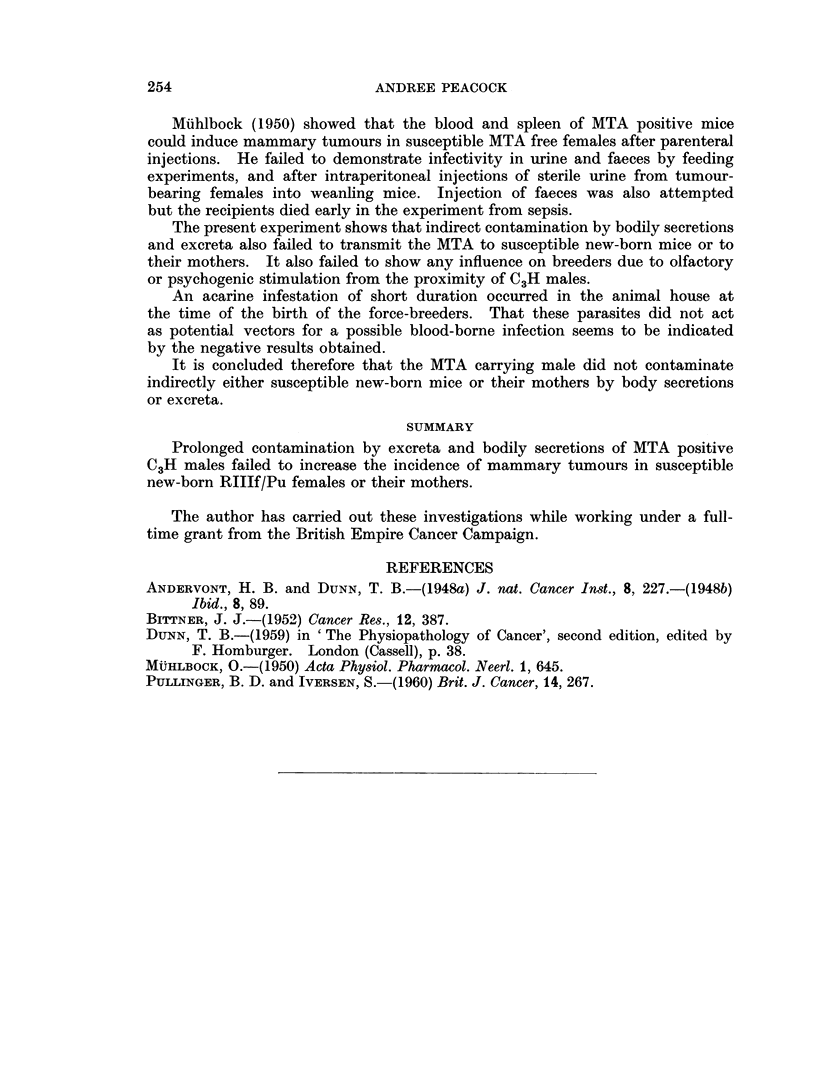# Attempts to Transfer the Mammary Tumour Agent from Infected Male Mice to Susceptible New-born Females

**DOI:** 10.1038/bjc.1963.35

**Published:** 1963-06

**Authors:** Andree Peacock


### ATTEMPTS TO TRANSFER THE MAMMARY TUMOUR AGENT FROM INFECTED MALE MICE TO SUSCEPTIBLE NEW-BORN FEMALES

ANDREE PEACOCK

*From the Cancer Research Department, Royal Beatson Memorial Hospital, Glasgow*

Received for publication April 30, 1963

THE mouse mammary tumour agent (MTA) in virus carrying strains is usually transmitted from mother to offspring while suckling the young. It is known to be harboured also by the males of the same strains in their reproductive organs and accessory glands (Andervont and Dunn, 1948*a*) from which it can be transmitted to virus susceptible females in the course of repeated mating to these same females and eventually to their offspring (Andervont and Dunn, 1948*b*) by the usual (milk) route (Bittner, 1952).

It was thought that the MTA might be associated with various excreta and body debris of the $C_3H$ (virus-positive) males and that the young susceptible mice exposed to these discarded products might acquire the mammary agent by oral, nasal or cutaneous contamination indirectly.

To test this possibility the following experiment was carried out.

### EXPERIMENTAL DATA

A virus free RIIIf/Pu male and one or two of its litter mate sisters were mated in the same cage and from this time spent their lives below a cage in which was housed a $C_3H$ virus carrying male. A wire netting floor allowed droppings to fall freely into the RIIIf/Pu cage directly below, but no contact was possible between $C_3H$ and RIIIf/Pu mice.

The RIIIf/Pu parents were left to breed and rear their families. The two sisters generally did not have their litters at the same time and the apparently non-pregnant female was temporarily withdrawn until her sister's litter had been weaned, three weeks later. These litters, once removed from their parents, were then force-bred, all sisters to one brother of the same litter.

### RESULTS

### (1) *Mothers*

Of 19 RIIIf/Pu (MTA negative) females mated with their brothers, only 1 developed a mammary tumour. This female, No. 19, which was killed at 577 days of age and had no living progeny, was the litter mate of female No. 18, which had amongst her progeny female No. 55 which developed a mammary tumour. The relationship is shown diagrammatically below :

No. 9

(litter mate sisters)

No. 18 (died at 776 days) — No. 19* (died at 577 days)

No. 55* (died at 556 days) — No progeny

* Indicates mammary tumour at time of death.

*Histology.*—No. 19 (Path. No. 51/62) had an adenocarcinoma of the third right mammary gland. The tumour consists of duct-like structures with walls several cells thick from which an undifferentiated adenocarcinoma has developed, invading the underlying muscle and lymphatics.

(2) *Female offspring*

Two RIIIf/Pu (MTA negative) females out of 60 offspring developed mammary tumours. One, No. 27, born from the second litter of mother No. 17 (not closely related to 18 or 19), was killed at 738 days, having herself given birth to two force-bred litters.

The other, No. 55 born from female No. 18's second litter, was killed at 556 days having given birth to three force-bred litters.

*Histology.*—No. 27 (Path. No. 1154/62) had an adenoacanthoma of the second right mammary gland with some adenocarcinomatous areas. It also had a lymphoma adjacent to the pancreas.

No. 55 (Path. No. 662/62) had an undifferentiated solid lobular tumour of the fifth right mammary gland with anaplastic areas.

These tumours were adenocarcinomas of anaplastic type, No. 19 bearing some resemblance to Dunn's (1959) type B tumour.

### DISCUSSION

From these results, it is evident that there has been no increase of mammary tumours in the young exposed to contamination from indirect male sources. The incidence is about 3 per cent on average for breeders of this strain not exposed to contamination (Pullinger and Iversen, 1960). The earliest death occurred at 108 days, but 40 out of 60 mice were still alive after the appearance of the first tumour at 556 days.

The 18 mothers of the force-breeders did not have any mammary tumours, though 6 of them lived longer than No. 19, which had no living progeny, but as stated above, developed a mammary tumour at 577 days.

The three tumours in this experiment were solitary tumours though in two cases (No. 27 and 55) probably of multifocal origin within the gland. They were randomly distributed between the possible mammary gland sites, and they occurred in middle or late life. These features are not typical of MTA induced tumours which often affect more than one mammary gland, are generally of uniform pattern, and occur before middle age.

Mühlbock (1950) showed that the blood and spleen of MTA positive mice could induce mammary tumours in susceptible MTA free females after parenteral injections. He failed to demonstrate infectivity in urine and faeces by feeding experiments, and after intraperitoneal injections of sterile urine from tumour-bearing females into weanling mice. Injection of faeces was also attempted but the recipients died early in the experiment from sepsis.

The present experiment shows that indirect contamination by bodily secretions and excreta also failed to transmit the MTA to susceptible new-born mice or to their mothers. It also failed to show any influence on breeders due to olfactory or psychogenic stimulation from the proximity of $C_3H$ males.

An acarine infestation of short duration occurred in the animal house at the time of the birth of the force-breeders. That these parasites did not act as potential vectors for a possible blood-borne infection seems to be indicated by the negative results obtained.

It is concluded therefore that the MTA carrying male did not contaminate indirectly either susceptible new-born mice or their mothers by body secretions or excreta.

### SUMMARY

Prolonged contamination by excreta and bodily secretions of MTA positive $C_3H$ males failed to increase the incidence of mammary tumours in susceptible new-born RIIIf/Pu females or their mothers.

The author has carried out these investigations while working under a full-time grant from the British Empire Cancer Campaign.

### REFERENCES

ANDERVONT, H. B. and DUNN, T. B.—(1948*a*) *J. nat. Cancer Inst.*, **8**, 227.—(1948*b*) *Ibid.*, **8**, 89.

BITTNER, J. J.—(1952) *Cancer Res.*, **12**, 387.

DUNN, T. B.—(1959) in ‘The Physiopathology of Cancer’, second edition, edited by F. Homburger. London (Cassell), p. 38.

MÜHLBOCK, O.—(1950) *Acta Physiol. Pharmacol. Neerl.* **1**, 645.

PULLINGER, B. D. and IVERSEN, S.—(1960) *Brit. J. Cancer*, **14**, 267.